# Phosphoproteomic Profiling Identifies Aberrant Activation of Integrin Signaling in Aggressive Non-Type Bladder Carcinoma

**DOI:** 10.3390/jcm8050703

**Published:** 2019-05-17

**Authors:** Barnali Deb, Vinuth N. Puttamallesh, Kirti Gondkar, Jean P. Thiery, Harsha Gowda, Prashant Kumar

**Affiliations:** 1 Institute of Bioinformatics, International Technology Park, Bangalore 560066, India; barnali@ibioinformatics.org (B.D.); vinuth@ibioinformatics.org (V.N.P.); kirti@ibioinformatics.org (K.G.); 2 Manipal Academy of Higher Education, Madhav Nagar, Manipal 576104, India; 3 School of Biotechnology, Amrita Vishwa Vidyapeetham, Kollam 690525, India; 4 Cancer Science Institute of Singapore, National University of Singapore, Centre for Translational Medicine NUS Yong Loo Lin School of Medicine, Singapore 117597, Singapore; 5 Comprehensive Cancer Center, Institut Gustave Roussy, 114 Rue Edouard Vaillant, 94800 Villejuif, France; 6 CNRS UMR 7057, Matter and Complex Systems, Université Paris Diderot, 10 rue Alice Domon et Léonie Duquet Paris, 75205 Paris, France

**Keywords:** urothelial cancer, phosphoproteomics, activated pathways, ingenuity pathway analysis, molecular subtypes

## Abstract

Bladder carcinoma is highly heterogeneous and its complex molecular landscape; thus, poses a significant challenge for resolving an effective treatment in metastatic tumors. We computed the epithelial-mesenchymal transition (EMT) scores of three bladder carcinoma subtypes—luminal, basal, and non-type. The EMT score of the non-type indicated a “mesenchymal-like” phenotype, which correlates with a relatively more aggressive form of carcinoma, typified by an increased migration and invasion. To identify the altered signaling pathways potentially regulating this EMT phenotype in bladder cancer cell lines, we utilized liquid chromatography-tandem mass spectrometry (LC-MS/MS)-based phosphoproteomic approach. Bioinformatics analyses were carried out to determine the activated pathways, networks, and functions in bladder carcinoma cell lines. A total of 3125 proteins were identified, with 289 signature proteins noted to be differentially phosphorylated (*p* ≤ 0.05) in the non-type cell lines. The integrin pathway was significantly enriched and five major proteins (TLN1, CTTN, CRKL, ZYX and BCAR3) regulating cell motility and invasion were hyperphosphorylated. Our study reveals GSK3A/B and CDK1 as promising druggable targets for the non-type molecular subtype, which could improve the treatment outcomes for aggressive bladder carcinoma.

## 1. Introduction

Advanced cancer therapeutics demands a detailed understanding of the altered mechanisms operating in malignant disease. Notably, in bladder carcinoma, the current treatment regimens and interventions are mostly determined by the clinicopathological characteristics of the tumor. Bladder carcinoma is categorized into two clinicopathologically distinct subgroups: non-muscle-invasive bladder cancer (NMIBC) and muscle-invasive bladder cancer (MIBC). At diagnosis, 75% of bladder carcinoma is NMIBC, whereas 20% to 25% is MIBC. About 50% to 70% of NMIBC, including Ta- and T1-stage tumors, frequently recur [1], whereas 10% to 15% progress to MIBC (T2, T3, and T4 stages) [2]. Unlike most other carcinomas, carcinoma in situ (Tis) tumors, a superficial tumor, progress very rapidly to MIBC. Emerging evidence suggests that tumor heterogeneity alters treatment response and confers resistance, leading to recurrence. Thus, a detailed understanding of the clinicopathological subgroups and their molecular alterations is of paramount interest, especially for targeted therapy. Identification of distinct molecular subtypes of NMIBC and MIBC could highlight clinically relevant signaling pathways.

Clinical staging is still not precisely defined for bladder carcinoma because of its highly heterogeneous nature. The advent of whole-genomic and transcriptomic approaches has transformed our understanding of cancer heterogeneity. Whole-genome sequencing and expression profiling, have uncovered molecular subtypes that relate to distinct clinical entities in various cancers. For instance, several studies have identified intrinsic subtypes of MIBC that resemble the intrinsic subtypes of breast cancer [3,4,5]. Comprehensive studies have defined the molecular subtypes in bladder carcinoma, mainly by analyzing gene expression data, specific mutations, and copy-number changes with recent analyses suggesting that specific genetic alterations in fibroblast growth factor receptor 3 (*FGFR3*), tumor protein P53 (*TP53*), retinoblastoma 1 (*RB1*), Erb-B2 receptor tyrosine kinase 2 (*ERBB2*), nuclear-factor, erythroid 2 like 2 (*NFE2L2*), and lysine demethylase 6A (*KDM6A/UTX*) are enriched in different molecular subtypes and are clinically actionable [6,7,8].

So far, the intrinsic subtypes of bladder carcinoma have been categorized into two to seven classes by different groups. A broad stratification of high-grade bladder carcinoma was described by the University of North Carolina (UNC), where the subtypes (luminal and basal-like) reflected the hallmarks of breast cancer [4]. This study classified luminal and basal subtypes of bladder carcinoma using a 47-gene signature. Another similar molecular classification (MD Anderson) using gene expression profiling, restricted to MIBCs, found three subgroups: basal, luminal, and p53-like MIBCs [5]; the inclusion of the “p53-like” subtype was based on the mRNA expression of p53, which can predict MIBC chemoresistance. The Cancer Genome Atlas (TCGA) research network also categorized the intrinsic subtypes of bladder carcinoma into four clusters (Clusters I–IV) based on an integrated analysis of the mRNA, miRNA, and proteome expression profiling of 129 tumor samples. However, the most extensive classification by LUND University was conducted on gene expression profiling data for 308 tumor tissue samples, and resulted in five intrinsic types of carcinoma, designated Urobasal A, Genomically unstable, Urobasal B, Squamous cell carcinoma-like, and Infiltrated [9]. The LUND molecular subtypes were further characterized into seven gene signature (subdividing Urobasal A and Genomically instable subtypes into two groups each), based on the differential expression of genes associated with biological processes [10]. Using a 117-gene signature, Hedegaard et al. [11] classified only NMIBCs into three subclasses (Class I–III) with basal- and luminal-like characteristics as well as clinical features. A later meta-analysis on the molecular subtypes (established by UNC, MDA, TCGA and LUND) revealed some consensus patterning among the molecular subtyping strategies [10]. However, there remain inaccuracies in the current staging systems, and the clinicopathological features may also influence treatment selection [12]. Such inadequacies are to be expected due to the low sample size in these studies and a large, collaborative study is needed to identify the molecular signatures before assigning treatment with conventional chemotherapy.

In a study by Warrick et al., bladder carcinoma cell lines were used to study the molecular subtypes in a relevant in vitro model. A detailed analysis was carried out on the publicly available data for the 27 bladder carcinoma cell lines (CCLE database) [13] as per the TCGA study. The panel of bladder carcinoma cell lines was classified into three subtypes based on gene expression clusters: luminal, basal, and “non-type” [14]. The non-type subtype displayed low expression of both luminal and basal markers. We further included these three subtypes for our deeper understanding of the molecular events involved in bladder carcinoma.

We employed liquid chromatography tandem-mass spectrometry (LC-MS/MS)-based approach to identify differentially expressed proteins in all three distinct subtypes. We also sought to evaluate pathways enriched in luminal, basal, and non-type subtypes. We undertook an extensive quantitative phosphoproteomic analysis of six bladder carcinoma cell lines (RT112, SW780, VMCUB-1, T24, J82, and UMUC3) and compared the phosphoproteomic data of bladder carcinoma cell lines with a non-neoplastic bladder cell line (TERT-NHUC) [15]. The six cell lines were previously assigned to luminal (RT112 and SW780), basal (VMCUB-1), and non-type (UMUC3, J82 and T24) molecular subtypes [14]. To our knowledge, this is the first phosphoproteomic profiling of bladder carcinoma and it may provide a global platform to identify the complex kinase-driven signaling events in bladder carcinoma.

## 2. Experimental Section

### 2.1. Cell Culture

Bladder carcinoma cell lines, SW780, RT112, VMCUB-1, T24, J82, and UMUC3 were cultured in dulbecco’s modified eagle’s medium (DMEM; with high glucose, HIMEDIA), supplemented with 10% fetal bovine serum (FBS) and 1% penicillin/streptomycin. Cells were maintained in a humidified incubator at 37 °C and 5% CO_2_. The non-neoplastic bladder cell line (TERT-NHUC) was cultured in KGM-Gold Media (Lonza Group, Allendale, NJ, USA) supplemented with hydrocortisone, transferrin, epinephrine, gentamicin, amphotericin B (GA-1000), bovine pituitary extract (BPE), recombinant human epidermal growth factor (rhEGF), and insulin in a humidified incubator at 37 °C and 5% CO_2_.

TERT-NHUC cells were kindly provided by Prof. M.A. Knowles, University of Leeds, UK [15].

### 2.2. Cell Lysis, Protein Extraction, and Digestion

Cell lines were grown to 70% confluence, starved in serum-free medium for 12 h, and then lysed in cell lysis buffer (2% SDS, 5 mM sodium fluoride, 1 mM β-glycerophosphate, 1 mM sodium orthovanadate in 50 mM triethyl ammonium bicarbonate (TEABC)). Protein concentration was estimated using the BCA method (Pierce; Waltham, MA, USA). Equal amount of protein from each cell line was used for protein digestion. Cell lysates were reduced and alkylated using 5 mM dithiothreitol (DTT) and 20 mM iodoacetamide (IAA), respectively. Samples were then digested overnight at 37 °C using trypsin (1:20) (Promega; San Luis Obispo, CA, USA).

### 2.3. TMT Labeling

Before labeling, peptides were reconstituted in 50 mM TEABC (pH 8.0). Equal amounts of peptides from each condition were used for 10-plex Tandem Mass Tag (TMT) labeling (Thermo Fisher Scientific, Rockford, IL, USA). Labeling was carried out as per the manufacturer’s protocol. TERT-NHUC was labeled with the channel 126, and SW780, RT112, VMCUB-1, T24, J82, and UMUC3 were labeled with 130N, 130C, 129C, 131, 128C, and 128N, respectively. The reaction was quenched by the addition of 8 µl of 5% hydroxylamine for 15 min at room temperature, and the samples were dried.

### 2.4. Basic pH RPLC (bRPLC) and TiO_2_-Based Phosphopeptide Enrichment

TMT-labeled peptides were pooled and fractionated using an Agilent 1100 high-pH reverse-phase liquid chromatography (RPLC) system with a flow rate of 1 mL/min, as described earlier [16]. Briefly, labeled peptides, resuspended in 1 mL of bRPLC solvent (10 mM TEABC pH 8.4), were fractionated using high-pH, reverse-phase XBridge C18 columns (5 µm, 250 × 4.6 mm; Waters Corporation, Milford, MA, USA), with an increasing gradient of bRPLC solvent B (10 mM TEABC in 90% ACN, pH 8.4). A total of 96 fractions were collected in a 96-well plate containing 0.1% formic acid. The fractions were then concatenated into 12 pools, vacuum-dried, and subjected to TiO_2_-based phosphopeptide enrichment [17]. The enriched phosphopeptides were eluted thrice into microfuge tubes with 40 µL of 2% ammonia solution containing 10 µL of 20% trifluoroacetic acid. The peptides were then dried, resuspended in 30 µL of 0.1% TFA, and desalted using C18 Stage Tips (Thermo Fisher Scientific). The eluted peptides were then subjected to LC-MS/MS analysis.

### 2.5. LC-MS/MS Analysis

Phosphoproteomic analyses were performed on an Orbitrap Fusion Tribrid mass spectrometer (Thermo Fisher Scientific) interfaced with an Easy-nLC II nanoflow liquid chromatography system (Thermo Fisher Scientific), as described earlier [18]. Briefly, each fraction was reconstituted in Solvent A (0.1% Formic acid) and loaded on trap column (75 µm × 2 cm) packed with Magic C18 AQ (Michrom Bioresources, Inc., Auburn, CA, USA). Peptides were resolved on an analytical column (75 µm × 15 cm) at a flow rate of 350 nL/min using a linear gradient from 5% to 60% ACN in a 120 min run. MS data were acquired using scan range of 400–1,600 m/z at mass resolution of 120,000, and MS/MS data were acquired using resolution of 30,000 at m/z of 400. HCD fragmentation for MS/MS analysis was carried out with isolation width of 2 m/z and normalized collision energy of 34%. Data-dependent acquisition was carried out where the most intense precursor ions were detected.

### 2.6. Data Analysis

The MS-derived data were searched using Mascot and Sequest HT search engines with Proteome Discoverer 2.0 (Thermo Fisher Scientific). Phosphopeptide-enriched fractions from each replicate were searched against the RefSeq protein database (version 70), National Institutes of Health, USA with carbamidomethylation of cysteine residues as a fixed modification. Oxidation of methionine; the phosphorylations of serine, threonine, and tyrosine; and the deamidation of asparagine and glutamine were selected as dynamic modifications. Trypsin was set as the protease and a maximum of one missed cleavage was allowed. Precursor mass tolerance was set to 20 ppm, and a fragment mass tolerance of 0.05 Da was allowed. All peptide-spectrum matches (PSMs) were identified at a 1% false-discovery rate (FDR). Posterior error probability was calculated for individual PSMs using a percolator, providing statistical confidence for each spectral match. The probability of phosphorylation for each site was calculated by the phosphoRS node in Proteome Discoverer. Only phosphopeptides with >75% site localization probability were considered for further analysis. A 2-fold cut-off was used for dysregulated phosphopeptides compared to TERT-NHUC cell line.

### 2.7. Scratch Wound Assay

For each cell line, 3 × 10^5^ cells were seeded into the wells of a 6-well plate. The experiment was conducted in triplicate. When the cells had reached 90% to 95% confluency, a scratch was made with a 20-μl pipette tip. Cells were washed gently with media, incubated with PBS, and then imaged. Cells were maintained in DMEM supplemented with 2% FBS. Cells were observed and imaged at 0 and 24 h. The rate of migration was calculated using ImageJ macros (v1.50i; National Institutes of Health, Maryland, USA). A *t*-test was used to calculate significance and the area covered by the migrating cells was plotted in a bar graph.

### 2.8. Invasion Assay

The transwell system was used to measure invasion, as reported earlier [19]. Briefly, 20,000 cells per 500μl of serum free media were seeded onto the Matrigel-coated PET membrane (BD Bio Coat Matrigel Invasion Chamber; BD Biosciences, CA, USA) in the upper compartment, while the lower compartment was filled with complete growth media. Plates were maintained at 37 °C for 48 h. After the incubation period, the upper membrane surface was wiped with a cotton-tip applicator to remove non-migratory cells. Cells that migrated to underside of membrane were fixed and stained using 4% methylene blue in 50% methanol. The number of cells that penetrated was counted for randomly selected viewing fields at 10× magnification using CX 41 Olympus Polarizing Trinocular microscope. The counting of cells was done using ImageJ multi-point cell counter (v1.50i; National Institutes of Health, Maryland, USA). The experiment was conducted in replicates.

### 2.9. Kinome Map

The kinome map was built using the KinMap tool (http://www.kinhub.org/kinmap/index.html). The list of identified kinases was searched and the relevant kinases are highlighted on the kinome map. Dysregulated kinases (hyper- or hypo-phosphorylated in any cell line) are also depicted.

### 2.10. Clustering the Molecular Subtypes

All quantified peptides (across all cell lines and in both replicates) were considered to identify differentially phosphorylated peptides. Fold-change was calculated by taking the intensity ratios with TERT-NHUC as the control. A *t*-test was used to determine the differentially phosphorylated peptides in the non-type subtype as compared with the luminal/basal subtype and the control cells. Differentially phosphorylated peptides (*p* ≤ 0.05) were further considered for supervised clustering using Perseus data analysis tool v1.5.8.5, Martinsried, Germany (http://www.biochem.mpg.de/5111810/perseuss) [20].

### 2.11. Ingenuity Pathway Analysis

Pathway analysis was completed for significantly dysregulated phosphopeptides in the non-type subtype using the Ingenuity Pathway Analysis tool (IPA Build: 460209M; Version: 39480507; IPA, Qiagen, Redwood City, CA, USA). The Core Analysis module in the IPA Ingenuity Knowledge Base reference repository was used to predict relationships of differentially phosphorylated molecules in our dataset. IPA was used to overlay our input dataset with that of the knowledge base. Canonical pathway analyses identified the top canonical pathways significantly enriched in our datasets. The “integrin pathway” was enriched in the total quantified dataset across all tested cell lines and with a confident phosphosite assignment. Significantly enriched functions and networks were also obtained. The IPA analysis results were schematically replicated using Adobe Illustrator (vCS5.1; Adobe Systems, San Jose, CA, USA).

### 2.12. Kinase-Substrate Enrichment Analysis

Kinase-substrate enrichment analysis was done using the online KSEA tool (https://casecpb.shinyapps.io/ksea/) [21]. Proteins with differentially phosphorylated sites in the non-type subtype were used for the input file, and analyzed using PhosphoSitePlus and NetworKIN as the background datasets. The *p*-value cutoff (for plot) and number of substrates cutoff were set to 0.05 and 5, respectively.

### 2.13. Motif Analysis

Motifs enriched in the non-type subtype were analyzed using the motif-x algorithm v1.210.05.06 (http://motif-x.med.harvard.edu). The “phospho-window” (7 amino acid residues on either side of the phosphorylated residue) was extracted from the RefSeq database. Significance threshold was set to *p* < 0.001. The minimum occurrence of the motif was set to 20 for pSer peptides and 10 for pThr against an IPI (International Proteome Index) human proteome background with the central character as “s” and “t”, respectively.

### 2.14. Immunohistochemistry

High-grade and low-grade bladder cancer FFPE tissue sections were obtained from Kidwai Institute of Molecular Oncology after informed patient consent. IHC was carried out on both the cases. Briefly, sections were deparaffinized and antigen retrieval was carried by incubating sections in antigen retrieval buffer (0.01 M Trisodium citrate buffer, pH 6) for 20 minutes. Endogenous peroxidases were quenched using (1:1) methanol: chloroform solution followed by washes with PBS plus 0.05% Tween-20. The sections were blocked using 5% goat serum to avoid non-specific binding of primary antibody for 30 minutes. Further sections were incubated with primary anti-Talin1 (S425) (Abcam, Cambridge, United Kingdom) antibody at 1:200 dilutions overnight at 4 °C in a humidified chamber. Next day, the sections were washed thrice with wash buffer and incubated with appropriate horseradish peroxidase conjugated rabbit secondary antibody for 30 minutes at room temperature. Excess secondary antibody was removed using wash buffer followed by addition of DAB substrate. The signal was developed using DAB chromogen (DAKO, Glostrup, Denmark) and counterstained by hematoxylin. The immunohistochemical labeling was assessed by an experienced pathologist. Images were taken at 10× on Olympus DP-21 microscope.

## 3. Results

### 3.1. EMT Scores of the Molecular Subtypes of Bladder Carcinoma Cell Lines

**Figure 1:**
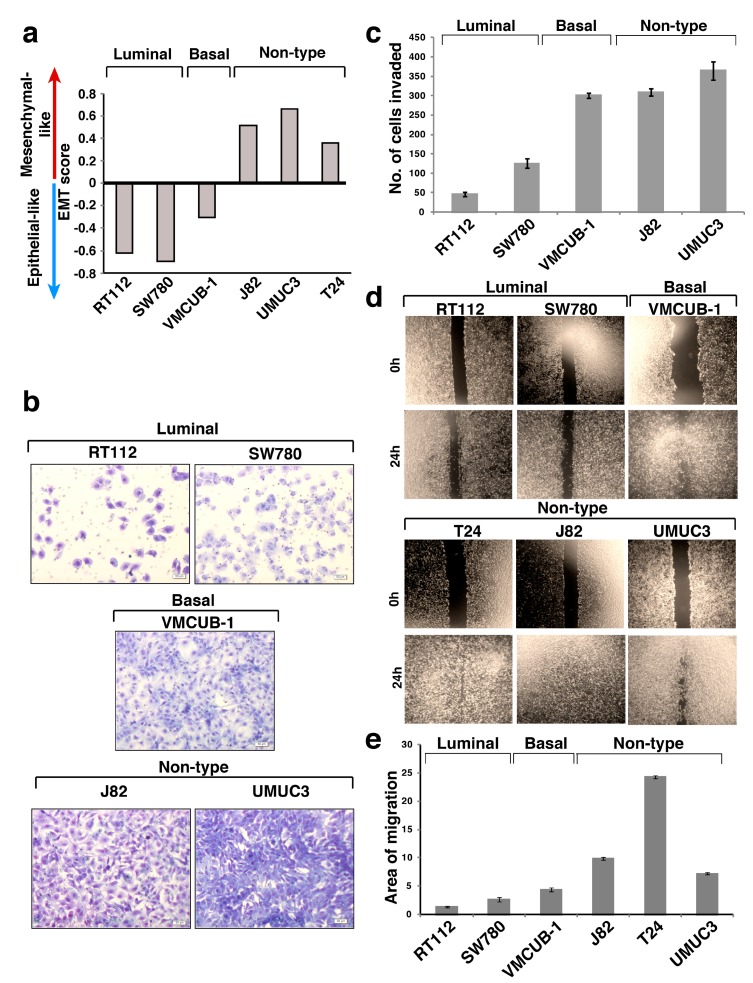
(**a**) Epithelial-mesenchymal transition score of the molecular subtypes of bladder carcinoma cell lines. (**b**) Invasion assay. Cells which invaded through the Matrigel were stained using methylene blue and imaged at 10× magnification. (**c**) Quantitative analysis of the number of cells which have invaded in each molecular subtype. (**d**) Scratch wound assay. Migration rate of the molecular subtypes of bladder carcinoma cell lines (magnification: 10×). (**e**) Quantitative analysis of scratch wound assay of the molecular subtype of bladder carcinoma.

EMT (−1.0 to +1.0) scores were computed to estimate the EMT phenotype of the molecular subtypes of bladder carcinoma cell lines (Supplementary Table S1) [3]. To compute the EMT score in bladder carcinoma cell lines, we adopted a similar approach to that used in ssGSEA [22]. Empirical cumulative distribution function (ECDF) was estimated for Epithelial and Mesenchymal gene sets. The 2KS test was employed to compute the difference between the Mesenchymal ECDF (ECDF_Mes_) and the epithelial ECDF (ECDF_Epi_). The EMT signature specific to bladder was curated and applied to single sample gene set enrichment analysis (ssGSEA) to provide a gross assessment for the EMT phenotype for each cell line. The BinReg EMT signature was used to predict the EMT phenotype in the cell lines [3,23]. A cell line with a positive EMT score exhibits a more “mesenchymal-like” phenotype, whereas a negative EMT score reflected a more “epithelial-like” phenotype. EMT scores suggested that non-type cell lines were “mesenchymal-like”, whereas the luminal and basal cell lines had an “epithelial-like” characteristic (Figure 1a).

### 3.2. Non-Type Bladder Carcinoma Has Increased Migration and Invasive Ability

We performed functional assays to determine and compare the tumorigenic properties of the non-type cell lines with the luminal/basal subtype. Using matrigel invasion assay, we estimated that the non-type were significantly more invasive as compared to the luminal (*p* = 6.9 × 10^−14^) and basal subtypes (*p* = 0.03) (Figure 1b,c). We found increased migration of non-type bladder carcinoma cell lines as compared to luminal (*p* = 0.002) and basal (*p* = 0.007) subtypes (Figure 1d,e).

### 3.3. Phosphoproteomic Analysis of Bladder Carcinoma Cell Lines

**Figure 2:**
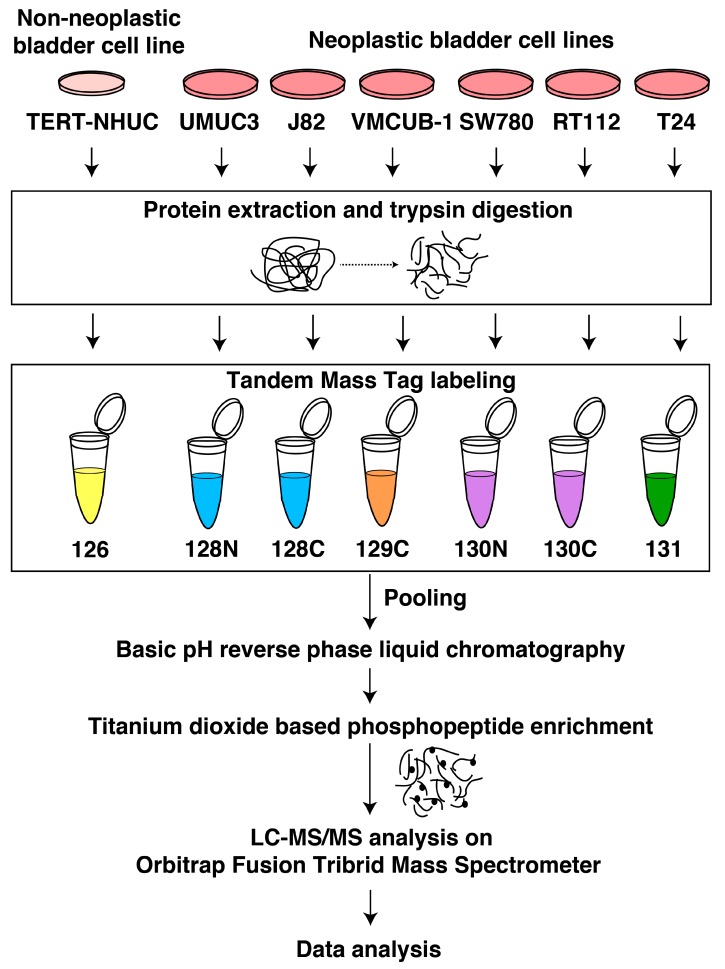
Workflow illustrating the quantitative phosphoproteomics analysis of bladder carcinoma cell lines. For sample processing, proteins were extracted from the bladder carcinoma and non-neoplastic cell lines and digested using trypsin. Each cell line was tagged using the tandem mass tags TMT labeling kit and lyophilized and enriched using the phosphopeptide enrichment protocol (titanium dioxide enrichment). The samples were run on Orbitrap Fusion Tribrid Mass Spectrometer and MS2-based quantitation was achieved. The files were searched against Mascot and Sequest HT search engines. PhosphoRS node was used for the phosphosite assignment. Data was acquired in technical replicates.

**Figure 3:**
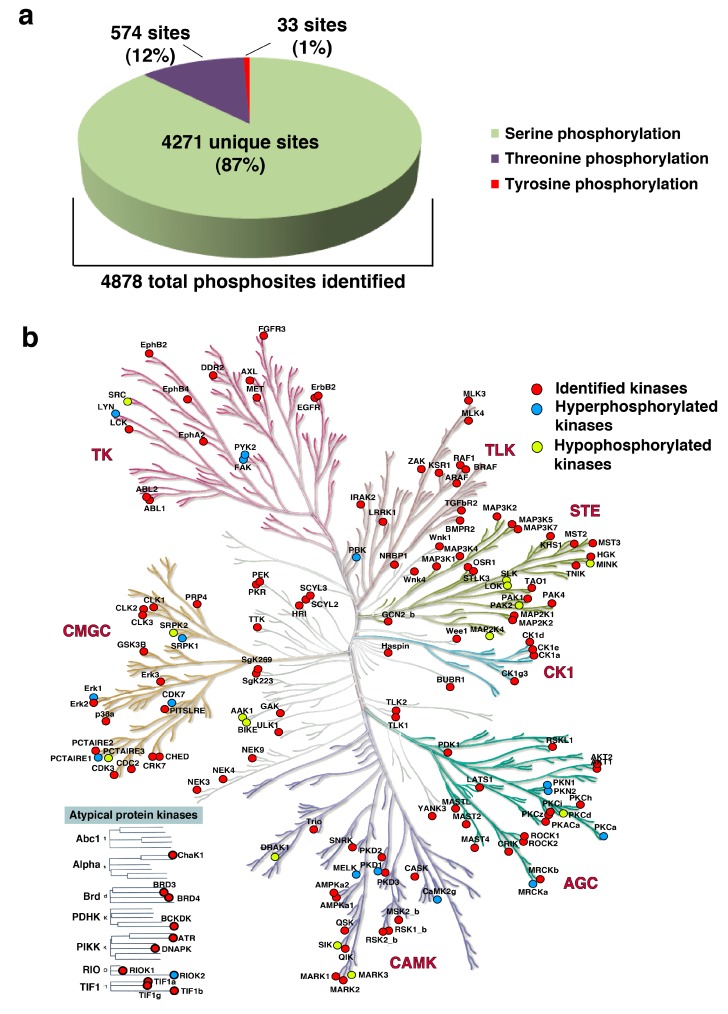
(**a**) Pie chart depicting the phosphosites identified in the study. Identification of the unique phosphosites (percentage of unique serine, threonine, and tyrosine sites identified in the study is mentioned). (**b**) Kinome map depicting the identified kinases in the dataset. Kinases are highlighted as in the inset legend. The map was built using the KinMap tool.

Phosphoproteomic profiling was conducted for six bladder carcinoma cell lines (RT112, SW780, VMCUB-1, J82, T24, and UMUC3) and the non-neoplastic bladder cell line (TERT-NHUC) using an established workflow for the enrichment of phosphopeptides (Figure 2). To increase the reliability of our phosphoproteomic analyses, we have included technical replicates in our study. The MS data were processed and searched against databases using SEQUEST-HT and MASCOT algorithms using the Proteome Discoverer 2.0 platform. Using an FDR cutoff of 1%, 10,979 phosphopeptides were identified, corresponding to 3125 proteins (Supplementary Table S2). The probability of Ser/Thr/Tyr phosphorylation sites on each peptide was calculated by the phosphoRS algorithm using a cut-off of >75%. We identified 4846 unique phosphopeptides corresponding to 2270 proteins (Supplementary Table S3), with a total of 4878 phosphosites identified: 4271 Ser residues, 574 Thr residues, and 33 Tyr residues (Figure 3a).

### 3.4. Kinases Enriched in the Phosphoproteomics Dataset

A total of 151 kinases were enriched in the phosphoproteomic analysis of the bladder carcinoma cell lines. Of these, 35 were CMGC (which refers to the CDK, MAPK, GSK3, and CLK set of families); 26 were of the homologs of yeast Sterile 7, Sterile 11, and Sterile 20 kinases (STE); 24 were from the protein kinase A, G, and C families (AGC); 20 were calmodulin/calcium regulated kinases (CAMK); 16 were tyrosine kinases (TK); 12 were tyrosine-like kinases (TLK); seven were casein kinases (CK1); and 11 were atypical kinases. In our dataset, 16 kinases were hyperphosphorylated and 14 were hypophosphorylated (in at least one cell line) (Figure 3b).

### 3.5. Unique Phosphorylation Signature Identified for the Non-Type Subtype of Bladder Carcinoma

In a recent detailed analysis, Warrick et al. used agglomerative methods (using the expression of a subtype-specific gene list from the TCGA study) to characterize molecular subtypes of bladder carcinoma cell lines [14]. Based on that report, we selected six bladder carcinoma cell lines to identify differential changes in the signaling pathways of the non-type molecular subtype (Table 1).

**Figure 4:**
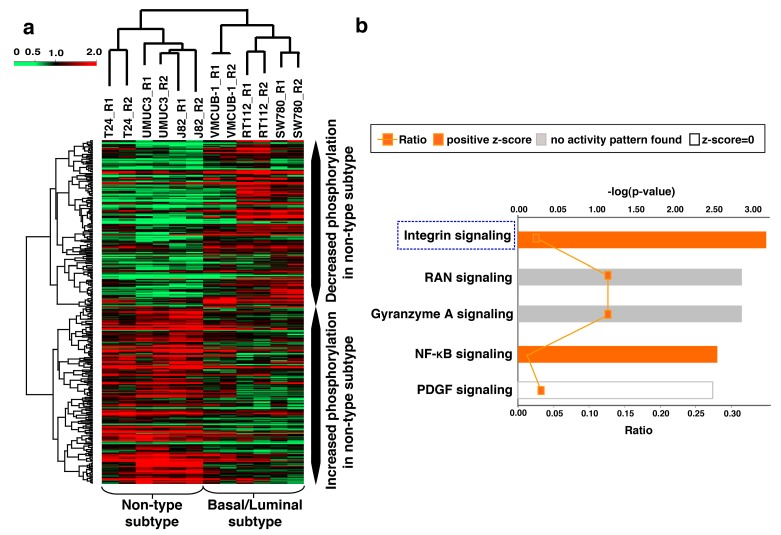
(**a**) Supervised clustering of the molecular subtypes of bladder carcinoma cell lines. A *t*-test conducted on the quantified data (across all cell lines) indicated that 375 peptides (corresponding to 289 proteins) were differentially phosphorylated in the non-type cell lines (T24, J82, and UMUC3) as compared with the luminal/basal subtype (SW780, RT112, and VMCUB-1) (*p* ≤ 0.05). (**b**) Canonical pathways enriched in the non-type subtype of bladder carcinoma. IPA analysis identified the “integrin pathway” as the most significantly enriched pathway in the aggressive non-type bladder carcinoma subtype.

Unsupervised hierarchical clustering was employed to cluster quantified phosphopeptides across normal and malignant bladder cell lines (Supplementary Table S4). The luminal subtype showed distinct clusters (RT112 and SW780), whereas the non-type cell lines (J82, UMUC3 and T24) were clustered along with the basal type (VMCUB-1) (Supplementary Figure S1). Thus, we sought to use the phosphorylation pattern or signature in the non-type subtype to explain what drives the mesenchymal phenotype. 375 differentially phosphorylated peptides corresponding to 289 proteins were identified in the non-type cell lines as compared to the luminal/basal subtype (*p* ≤ 0.05) (Supplementary Table S5). Supervised clustering of the 375 differentially phosphorylated peptides revealed distinct phosphopeptide signature. Two distinct clusters were observed: the non-type cell lines were clustered away from the luminal/basal cells (Figure 4).

### 3.6. Ingenuity Pathway Analysis (IPA) Identifies Aberrant Activation of Pathways in the Molecular Subtypes of Bladder Carcinoma

**Figure 5:**
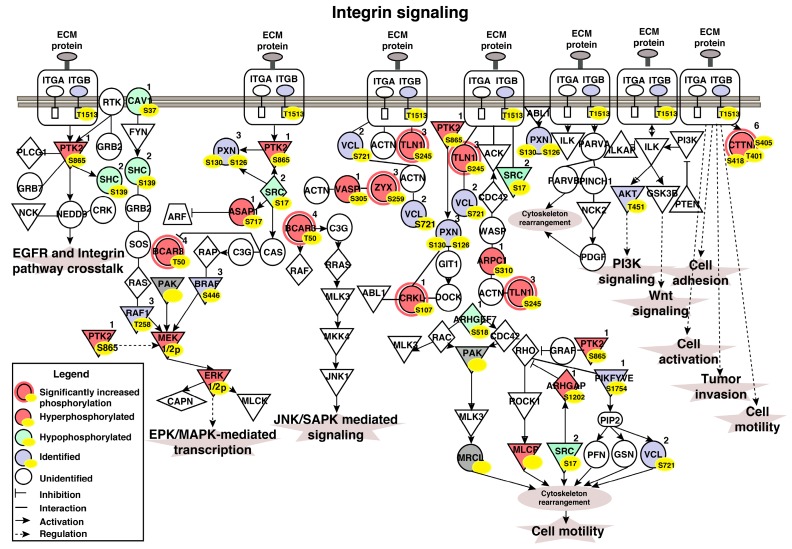
Schematic diagram of enriched integrin signaling pathway in non-type subtype of bladder carcinoma cell lines. Ingenuity pathway analysis lead to the identification of integrin signaling to be most enriched in the aggressive molecular subtype. The dysregulated proteins, the number of phosphopeptides, and the phosphosites identified are highlighted in the pathway.

IPA of 46 hyperphosphorylated peptides identified significant enrichment of “integrin pathway” components (*p* = 6.29E-04; *z*-score = 2.00) (Figure 4b). Five proteins involved in cell motility had significantly increased phosphorylation in the non-type subtype as compared with the basal and luminal subtypes as well as the TERT-NHUC (control) cells (Supplementary Table S6). TLN1 was 3-fold hyperphosphorylated in the non-type and 2-fold in the basal. We further checked the hyperphosphorylation of TLN1 (S425) in bladder tumor sections. Immunohistochemical staining showed hyperphosphorylation of TLN1 (S425) in high-grade tumors as compared to low-grade tumors (Supplementary Figure S2). TLN1 is a major mediator of the integrin activation and crosstalk. It also stabilizes extracellular matrix-actin linkage [24]. CTTN and CRKL were 1.5-fold hyperphosphorylated in the non-type subtype. CTTN phosphorylation is reported to mediate and accompany integrin mediated cell adhesion to the extracellular matrix [25]. The translocation of CRKL to the focal adhesion activates integrin induced downstream signaling through Src family of kinases and further mediates cell migration [26]. ZYX and BCAR3 were 1.5-fold hyperphosphorylated in the non-type subtype whereas 0.5-fold hypophosphorylated in the luminal subtype. ZYX regulates cellular movement and binds strongly to the focal adhesions. Integrins bind its cytoplasmic tail to ZYX through alpha-actinin [27]. BCAR3 along with its binding partner BCAR1 leads to changes in cellular morphology, motility, and adhesion by the transduction of integrin signaling [28]. To check the global phosphorylation status in aggressive non-type we carried out a pathway analysis with the set of all the phosphopeptides quantified across all cell lines (2806 phosphopeptides). The analysis displayed integrin pathway enrichment which comprises global proposition of the other proteins (the other proteins which are identified/dysregulated in our study) (*p* = 5.06E-04; *z*-score = 5.303) with the total inclusion of 49 proteins in the signaling network (Figure 5).

For the luminal subtype, there was an enrichment of “phospholipase C signaling” components (*p* = 6.29E-03; *z*-score = 2.00), including four differentially phosphorylated proteins—Rho guanine nucleotide exchange factor 16 (AREGEF16), actin-related protein 2/3 complex subunit 1B (ARPC1B), cdc42 effector protein 1 (CDC42EP1), and stathmin1 (STMN1). The basal subtype showed enrichment of “signaling by Rho family GTPase” components (*p* = 3.68E-03; *z*-score = 2.00), and included the differential phosphorylation of neuroblast differentiation-associated protein (AHNAK), AREGEF16, myristoylated alanine-rich C-kinase substrate (MARCKS), and proto-oncogene tyrosine-protein kinase Src (SRC) (Supplementary Figure S3).

### 3.7. Regulatory Interaction Network Enriched in the Non-Type Subtype

Network analysis appeared to link key molecules not identified in our study which may be important regulators of the network along with the non-type subtype specific phosphoproteins. The most enriched network in the non-type subtype contained 25 proteins from our dataset (score = 46) (Supplementary Figure S4a). The primary hub proteins included protein kinase A catalytic subunit (PRKACA), Rac-alpha serine/threonine protein kinase (AKT) and nuclear factor NF-kappa-B (NFκB). PRKACA interacts with protein kinase A regulatory subunit 1A (PRKAR1A), ADP ribosylation factor guanine nucleotide exchange factor 2 (ARFGEF2) and A-kinase anchor protein (AKAP11). AKT interacts which TBC2 domain family 4 (TBC2D4) and interferon-induced, double-stranded RNA activated protein (EIF2AK2), desmoplakin (DSP), SRC, and integrin beta-4 (ITGB4). NFκB interacts with TGFβ-activated kinase 1 (TGFB1), MAP3K7-binding protein 2 (TAB2), TBC2D4, casein kinase II subunit beta (CSNK2B), EIF2AK2, CDC42, CDC42EP1, DNA polymerase alpha subunit B (PLOA2), sentrin-specific protease 1 (SENP1), PKA, and immunoglobulin G (IgG). Cell motility was the most enriched function determined by the IPA analysis. 21 proteins related to cell motility were enriched in the non-type subtype of bladder carcinoma (Supplementary Figure S4b).

### 3.8. CDK1 (Cyclin-Dependent Kinase 1) and GSK3A/GSK3B (Glycogen Synthase Kinase 3A and 3B) Are the Predicted Activated Kinases in Non-Type Subtype of Bladder Carcinoma Cell Lines

**Figure 6:**
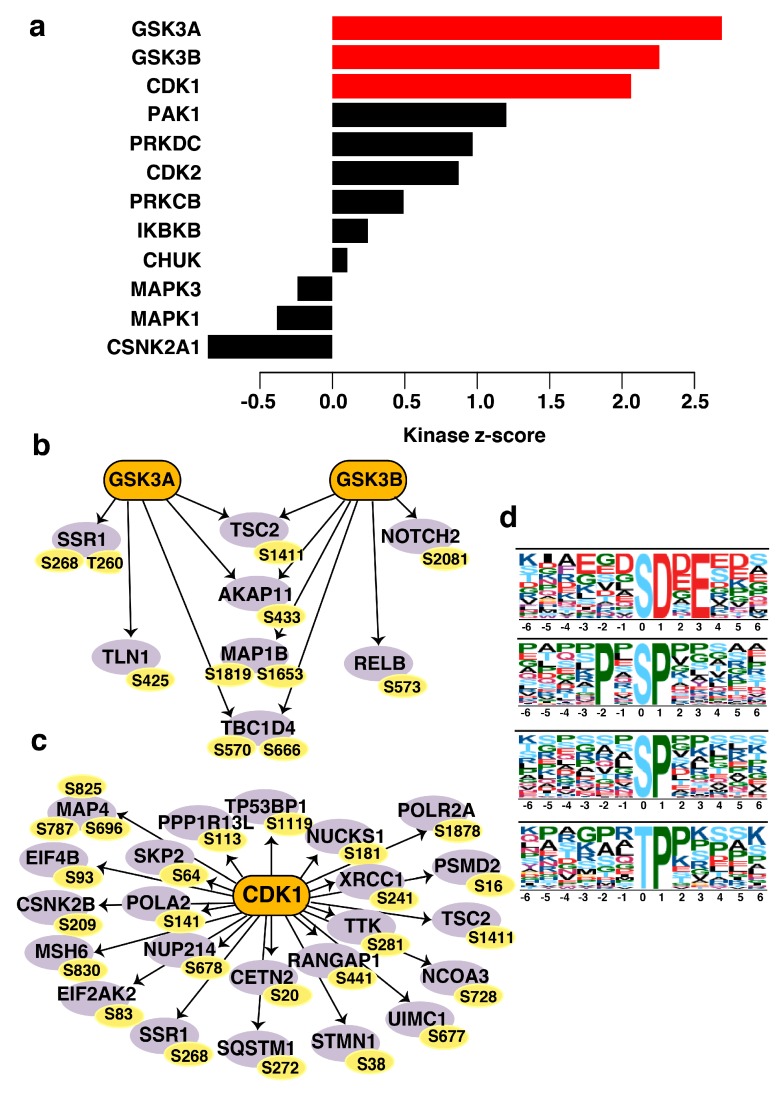
(**a**) Predicted upstream kinases enriched in the non-type subtype of bladder carcinoma cell lines. Graph showing the positively regulated upstream kinases (red bars) predicted to be activated in the non-type subtype. (**b**) Substrates of GSK3A/3B and (**c**) CDK1 enriched in non-type molecular subtype depicted by a schematic diagram. The respective phosphosites of the substrates identified are also highlighted. (**d**) Motif analysis of differentially phosphorylated peptides of non-type subtype bladder carcinoma. Serine and threonine motifs identified in the non-type subtype of bladder carcinoma.

Eight kinases—GSK3A, GSK3B, CDK1, ribosomal protein S6 kinase alpha-1 (RPS6KA1), ribosomal protein S6 kinase alpha-1 (RPS6KB1), serine/threonine protein kinase Sgk 1 (SGK1), TGFβ receptor type-2 (TGFBR2), and 5′-AMP-activated protein kinase catalytic subunit alpha-2 (PRKAA2)—were predicted to be significantly enriched (Supplementary Table S7). GSK3A (*p* = 0.003; *z*-score = 2.7) and GSK3B (*p* = 0.01; *z*-score = 2.3) were predicted to be activated and responsible for the phosphorylation of four downstream proteins (tuberin (TSC2), AKAP11, MAP1B, and TBC1 domain family 4 (TBC1D4)). GSK3A was predicted to independently phosphorylate translocon-associated protein subunit alpha (SSR1), whereas GSK3B was predicted to be upstream of neurogenic locus notch homolog protein 2 (NOTCH2) and transcription factor RelB (RELB). CDK1 was also predicted as the upstream activated kinase (*p* = 0.01; *z*-score = 2.06) for 23 proteins at single, double, or triple phosphorylation sites (Supplementary Table S8). RPS6KA1, RPS6KB1, and SGK1 were predicted as the negatively regulated kinases (*p* = 0.01; *z*-score = −2.10) (Figure 6a–c).

### 3.9. Active Proline-Directed Motifs Identified in the Non-Type Molecular Subtype

In the non-type subtype, five serine and one threonine phosphorylated motifs were significantly differentially phosphorylated (among the 289 signature proteins). A maximum fold change of 36 was calculated for the pSDxE serine phosphorylated motif. The consensus motifs PxpSP and pSP motifs also showed a high fold change in phosphorylation of 7.5-fold. Supplementary Table S9 enlists the differentially phosphorylated protein sites matching to the consensus motifs. Only one motif for the threonine sites (pTP) was identified in the dataset (10-fold enriched among 24 phosphopeptides) (Figure 6d).

## 4. Discussion

A molecular subtype is an “intrinsic” feature that defines both clinical and biological stratification of a given tumor. The molecular subtypes of bladder carcinoma have not been extensively studied. In addition, switching between molecular subtypes and the multifocal characteristics of these tumors are two phenomena that preclude our understanding of the biological and clinical properties of these tumors. Most of the studies have defined the molecular subtypes by analyzing gene expression data based on the enrichment of specific genetic alterations and gene expression profiles. However, a comprehensive and quantitative phosphoproteomics study on bladder carcinoma has still not been reported. Here we provide compelling data that suggests the cellular signaling pathways that contribute to the mesenchymal phenotype in the non-type molecular subtype of bladder carcinoma. Our study identified 10,979 phosphopeptides corresponding to 3125 proteins from a panel of bladder carcinoma cell lines. This study offers new biological insight and identifies potential kinases that could be targeted in bladder carcinoma. We combined bioinformatics approaches with phosphoproteomic analyses of bladder carcinoma cell lines to generate highly enriched cellular signaling pathways. This provided the groundwork for defining the molecular subtypes in bladder carcinoma cell lines, which may help to identify and develop new therapeutic approaches within these model systems. Importantly, the study needs to be extended on large cohort of bladder tumor sections to validate a prognostic and predictive role of the subtype specific signature. This would aid in the clinical assessment of bladder carcinoma patients for appropriate treatment regimen based on the molecular subtypes. The computed EMT score suggests that the non-type molecular subtype is more “mesenchymal-like,” whereas the luminal/basal subtypes are “epithelial-like.” The non-type subtype cell lines show an increased migratory and invasive phenotype, reflecting typical characteristics of a mesenchymal-like phenotype. Non-type carcinomas may; thus, more readily invade the bladder wall, and this knowledge may provide the necessary evidence to spur a change in the field of muscle invasive bladder carcinoma, particularly in terms of their recurrent nature. Recently, existence of hybrid EMT state has also been reported by various groups [29,30]. A study by Jolly et al. showed that hybrid epithelial/mesenchymal cells adhere higher tumor-initiation capabilities and metastatic potential [31]. Another study by Yadavalli et al. computed the EMT score using transcriptomic datasets. The EMT scores distributed between −0.3 to +0.5, for cancer CTCs suggestive of intermediate phenotypes [32]. In our study, we observed the distribution of EMT scores in the aggressive non-type subtype were distributed between +0.35 to +0.65.

We identified the enrichment of the integrin signaling pathway in the non-type molecular subtype, with differential phosphorylation in five major proteins: TLN1, CTTN, BCAR3, CRKL, and ZYX. Integrins are a family of cell adhesion receptors that mediate cell–matrix and cell–cell interactions [33,34,35]. A major function of the integrins is to regulate the intracellular signaling cascades that lead to cell proliferation, survival, motility, and migration [36,37,38]. TLN1 is an adaptor protein critical to integrin signaling. In the non-type molecular subtype, TLN1 is significantly hyperphosphorylated at S425 in the head domain (band 4.1, ezrin, radixin, moesin homology domain) (Supplementary Figure S5), a site also known to be hyperphosphorylated in prostate cancer [39]. TLN1 S425 is phosphorylated by cyclin-dependent kinase 5 (CDK5), which controls the metastatic potential in prostate cancer [40]. S425 acts on integrin β with an “inside-out” mechanism; however, we did not identify a change in integrin β1. Cell migration is stimulated by TLN1 coupled to the integrin cytoplasmic domain: TLN1 connects ligand-bound integrins with the actin cytoskeleton, and this is needed for the catalysis of the focal adhesion-dependent pathways [41] to initiate cell movement. The phosphorylation of TLN1 at S425 limits focal-adhesion turnover, which, in turn, stabilizes the lamellipodia contributing to the sustained cell migration [42]. TLN1 overexpression is also correlated with advanced and aggressive oral squamous cell carcinoma [43]. In concordance with the reported studies, we also observed a hyperphosphorylation of TLN1 (S425) in high grade tumor sections. In our unpublished global proteomics data of bladder carcinoma cell lines, TLN1 is >1.5-fold overexpressed in the non-type cell lines; however, its role in bladder carcinoma and whether it contributes to the “mesenchymal-like” phenotype is unclear. CTTN is an actin-binding protein significantly hyperphosphorylated at S418, S405, and T401 in the non-type molecular subtype. CTTN promotes cell migration by lengthening lamellipodia, increasing the number of filopodia, and increasing protrusion time [44]. CTTN is phosphorylated at S418 and S405 by the serine/threonine extracellular signal-regulated kinases (ERK1/2) [45], which, in turn, stimulates binding of the Arp2/3 complex to N-WASP via its SH3 domain [46]. CTTN phosphorylation is required for actin regulation and is responsible for lamellipodia formation and associated with enhanced cancer cell migration [47]. In bladder carcinoma, CTTN may be involved in the regulation of actin-based extravasation through invadopodia formation [48]. CRKL is another adaptor protein that is overexpressed in many cancers, including bladder carcinoma [49]. It regulates cyclin D1 and modulates ERK1/2 phosphorylation. In bladder carcinoma cell lines, CRKL is hyperphosphorylated at S107; however, the function of this phosphorylation is unclear. The two other differentially phosphorylated proteins identified in our study are ZYX and BCAR3. ZYX is a LIM domain protein with distinct actin polymerization activity. It also modulates cell adhesion and the expression of integrins, controlling cell motility in lung carcinoma. ZYX may also regulate EMT during lung cancer development and may regulate cell–cell adhesion, integrin α5β1 expression, and ECM adhesion [50]. ZYX is overexpressed in breast cancer and positively correlated with breast carcinoma metastasis [51]. Its depletion reduces cell proliferation, motility, and in vivo tumorigenic activity. BCAR3, on the other hand, binds to the C-terminal of its partner p130Cas (BCAR1) and co-ordinates the activation of SRC, a non-receptor tyrosine kinase [52]. The BCAR3–SRC activation axis is governed by PTPα, binding to its SH2 domain to recruit the BCAR3/Cas complex. T789 phosphorylation of PTPα is required for integrin induced-adhesion signaling [53]. The functional roles of ZYX (S259) and BCAR3 (T50) phosphorylation have not been studied in bladder carcinoma, but these proteins may contribute to invasion, as both proteins are involved in cell motility. However, EMT is regulated at several levels through transcriptional controls, epigenetic modifications, translational modifications, and alternative splicing [54,55,56,57,58].

Phospholipase C has a decisive role in skin cancer development [59], and its overexpression in gastric-mucosa cells perhaps offers an opportunity to differentiate gastric cancer and inflammatory lesions [60]. Thus, its overexpression in luminal bladder carcinoma is not unexpected, and may be the result of early pro-inflammatory signaling. Our pathway analysis also identified an enrichment of Rho signaling in the basal cell line. Activated Rho proteins can alter cell behavior as well as cell morphology. In breast cancer, Rho family GTPases regulates cell motility by cytoskeleton remodeling and altering focal adhesions [61]. High RhoA and RhoC expression is correlated with poor tumor differentiation, muscle invasion, and lymph node metastasis in bladder carcinoma [62]. The basal subtype of bladder carcinoma is relatively more aggressive than the luminal subtype, and activated Rho protein signaling may contribute to this aggressive clinical outcome [63].

In our IPA analysis, NFκB was highlighted as the regulatory hub molecule of the most enriched network in the non-type subtype. This transcriptional regulator largely affects the expression of cytokines, chemokines, adhesion molecules, and controls various mechanisms, including inflammation, proliferation, transformation, angiogenesis, invasion, and chemoresistance [64]. Interestingly, β1-integrin contains a unique NFκB-binding site in its promoter region, suggesting a potential regulatory role of NFκB in integrin signaling [65]. Thus, NFκB may regulate the expression of integrins and enhance signaling downstream, affecting cell proliferation and motility.

The non-type significantly enriched in golgi vesicle (0.09%), cytoskeleton (5.1%) and in nuclear compartment (75.2%). In addition, ubiquitin-specific proteases (4.9%), cytoskeleton protein binding (4.5%), and signal transducers (1.1%) were significantly active in non-type bladder carcinoma cell line (Supplementary Figure S6). Through our kinase-substrate enrichment analysis, we predicted that the two most activated kinases were GSK3A and GSK3B, two highly conserved serine/threonine protein kinases. The roles of these two kinases in cancer remain controversial, with little evidence in the literature to support their role in the disease. The Y216-phosphorylated active form of GSK3B is significantly decreased in squamous cell carcinoma [66] and in lung cancer, inhibiting GSK3B increases the expression of the transcriptional repressor Snail, which suppresses E-cadherin and promotes EMT [67]. A potent GSK3 inhibitor LY2090314 has been tested in clinical trials against metastatic pancreatic cancer (NCT0163230) and acute leukemia (NCT01214603) [68]. Others have reported that pharmacological inhibition of GSK3B can induce apoptosis by sorafenib in melanoma cell lines and leukemic cells [69,70]. Yet, in ovarian cancer, GSK3B activity was linked with cell proliferation, and overexpression of its active form can induce CDK1 expression and facilitate cell proliferation [71]. CDK1 is a major mitotic regulating kinase involved in the G2/M phase of the cell cycle, and is upregulated in several cancers and also associated with clinicopathological factors [72,73,74]. Various CDK inhibitors have been trialed for the treatment of breast cancer and chronic lymphocytic leukemia [75,76,77]. Nevertheless, the role of CDK1 in bladder carcinoma has yet to be elucidated. GSK3A/B and CDK1 could be potential druggable targets for the aggressive non-type bladder carcinoma. Intriguingly, we identified enrichment of proline-directed motifs (pSP/ pTP) in our dataset, which are known to be active mostly in proliferating cells and phosphorylated by CDK1 during mitosis [78]. We also identified the pSer and pThr motifs that recognize acidic casein kinase II in our dataset [79], yet the importance of these motifs in bladder carcinoma remains unclear.

## 5. Conclusions

We present the first comprehensive phosphoproteome of bladder carcinoma cells and offer a potential starting point for further mechanistic studies. Our phosphoproteomic data and bioinformatic analyses suggest two key activated kinases (GSK3A/B and CDK1) as potential druggable targets for the aggressive subtype of bladder carcinoma. We provide a glimpse of the dynamic process of phosphorylation in bladder carcinoma and also the differentially regulated phosphoproteins in this aggressive molecular non-type subtype.

## Figures and Tables

**Table 1:** General characterization and molecular subtypes of the bladder carcinoma cell lines.

Cell Line	Source ^a^	Molecular Subtype ^b^	Derived from Male/Female	Grade
SW780	UCC	Luminal	Female	Grade 1
RT112	UCC	Luminal	Female	Grade 2
T24	EC	Non-type	Female	Grade 3
J82	EC	Non-type	Male	Grade 3
UMUC3	UCC	Non-type	Male	-
VMCUB-1	EC	Basal	Male	Grade 2

^a^ UCC, urothelial cell carcinoma; EC, epithelial carcinoma; ^b^ Molecular subtypes are from Warrick et al., 2016 [14].

## Supplementary Materials

The following are available online at https://www.mdpi.com/2077-0383/8/5/703/s1, Figure S1: Unsupervised clustering of the bladder carcinoma cell lines of the quantified data across all the cell lines; Figure S2: Immunohistochemistry showing TLN1 (S425) hyperphosphorylation in high-grade tumors as compared to low-grade tumors; Figure S3: Top five canonical pathways enriched by IPA in (a) luminal and (b) basal subtypes of bladder carcinoma; Figure S4: (a) A global network interaction map of the differentially phosphorylated proteins in the non-type subtype. Most enriched network in the aggressive subtype with an enrichment score of 46 (IPA). (b) Cell motility is an intrinsic characteristic of the non-type subtype of bladder carcinoma. Phosphoproteins involved in cell motility, as depicted from the IPA analysis; Figure S5: Protein domain structure for TLN1. The domains of TLN1 and the significantly differentially phosphorylated sites (S425; head domain) in the non-type cell lines; Figure S6: Gene ontology enrichment of the differentially phosphorylated proteins in the non-type molecular subtype. Most of the proteins are nuclear proteins (75.2%). Proteins with kinase activity and signal transducer activity are also significantly enriched (*p* ≤ 0.05). Data were analyzed using FunRich; Table S1: EMT score of bladder carcinoma cell lines molecular subtypes; Table S2: List of phosphopeptides identified in bladder carcinoma cell lines using SequestHT and Mascot search algorithms; Table S3: List of phosphopeptides identified in bladder carcinoma cell lines using SequestHT and Mascot search algorithms with a PhosphoRS probablity of ≥75%; Table S4: List of phosphopeptides identified in bladder carcinoma cell lines using SequestHT and Mascot search algorithms with a PhosphoRS probability of ≥75% and quantified across all the cell lines; Table S5: A total of 375 differentially phosphorylated peptides in the non-type bladder carcinoma molecular subtype; Table S6: Proteins differentially phosphorylated in non-type subtype in integrin signaling (*p*-value ≤ 0.05); Table S7: Kinase scores for the kinase-substrate enrichment analysis; Table S8: Kinase-substrate enrichment scores; and Table S9: Motifs identified by motif-x in the differentially phosphorylated peptides in non-type subtype.
