# Supplementary material for: Phosphoproteomic Profiling Identifies Aberrant Activation of Integrin Signaling in Aggressive Non-Type Bladder Carcinoma
Source: J Clin Med. 2019 May 17;8(5):703. doi: 10.3390/jcm8050703 (PMC6572125; doi:10.3390/jcm8050703)
Supplement: Supplementary file 1 [file jcm-08-00703-s001.zip › Deb et al,_Supplementary figures_JCM_032719.pdf]

Phosphoproteomic profiling identifies aberrant activation of integrin signaling in aggressive non-type bladder carcinoma

Barnali Deb, Vinuth N. Puttamallesh, Kirti Gondkar, Jean Paul Thiery, Harsha Gowda, Prashant Kumar

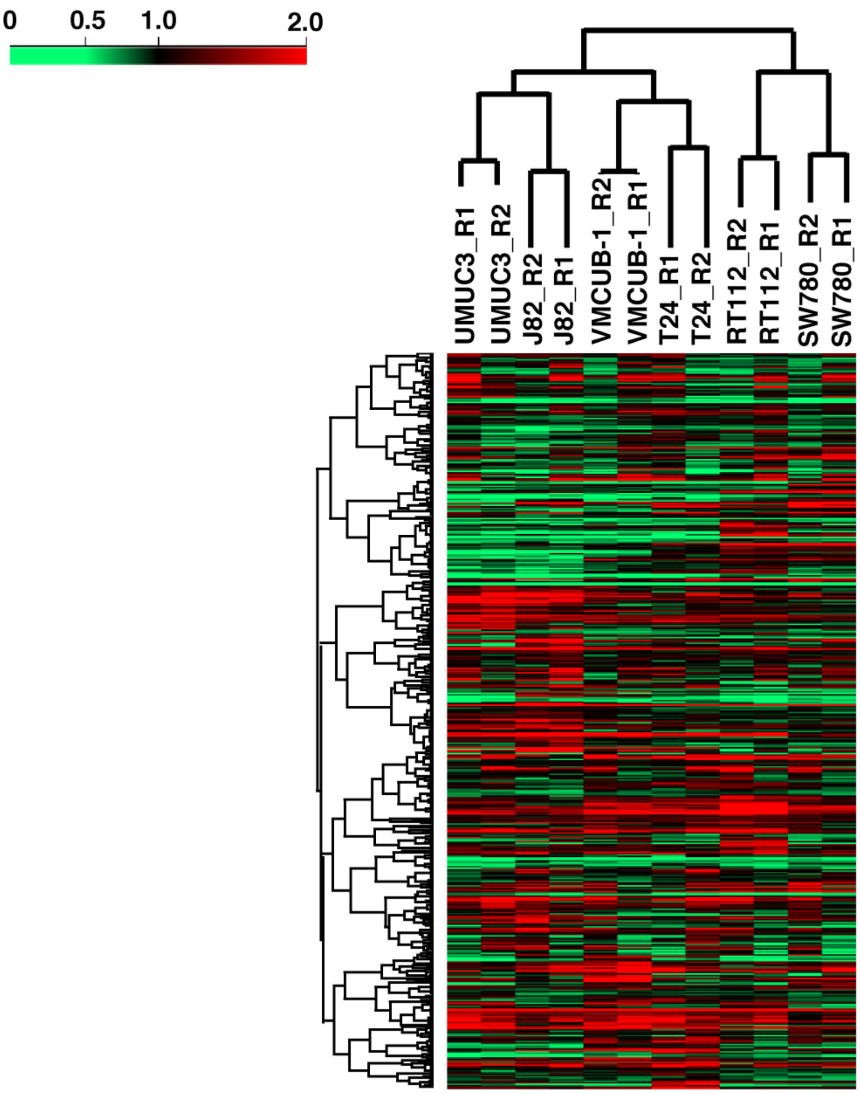

Supplementary Figure S1. Unsupervised clustering of the bladder carcinoma cell lines of the quantified data across all the cell lines.

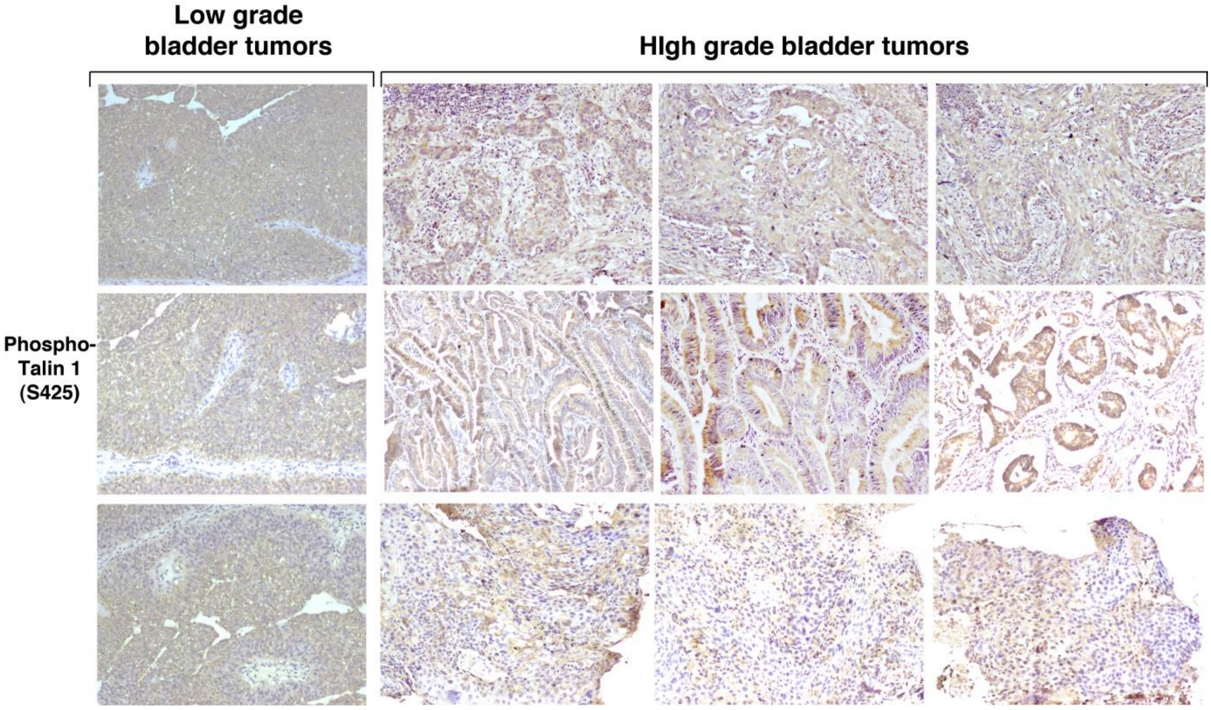

Supplementary Figure S2. Immunohistochemistry showing TLN1 (S425) hyperphosphorylation in high-grade tumors as compared to low-grade tumors.

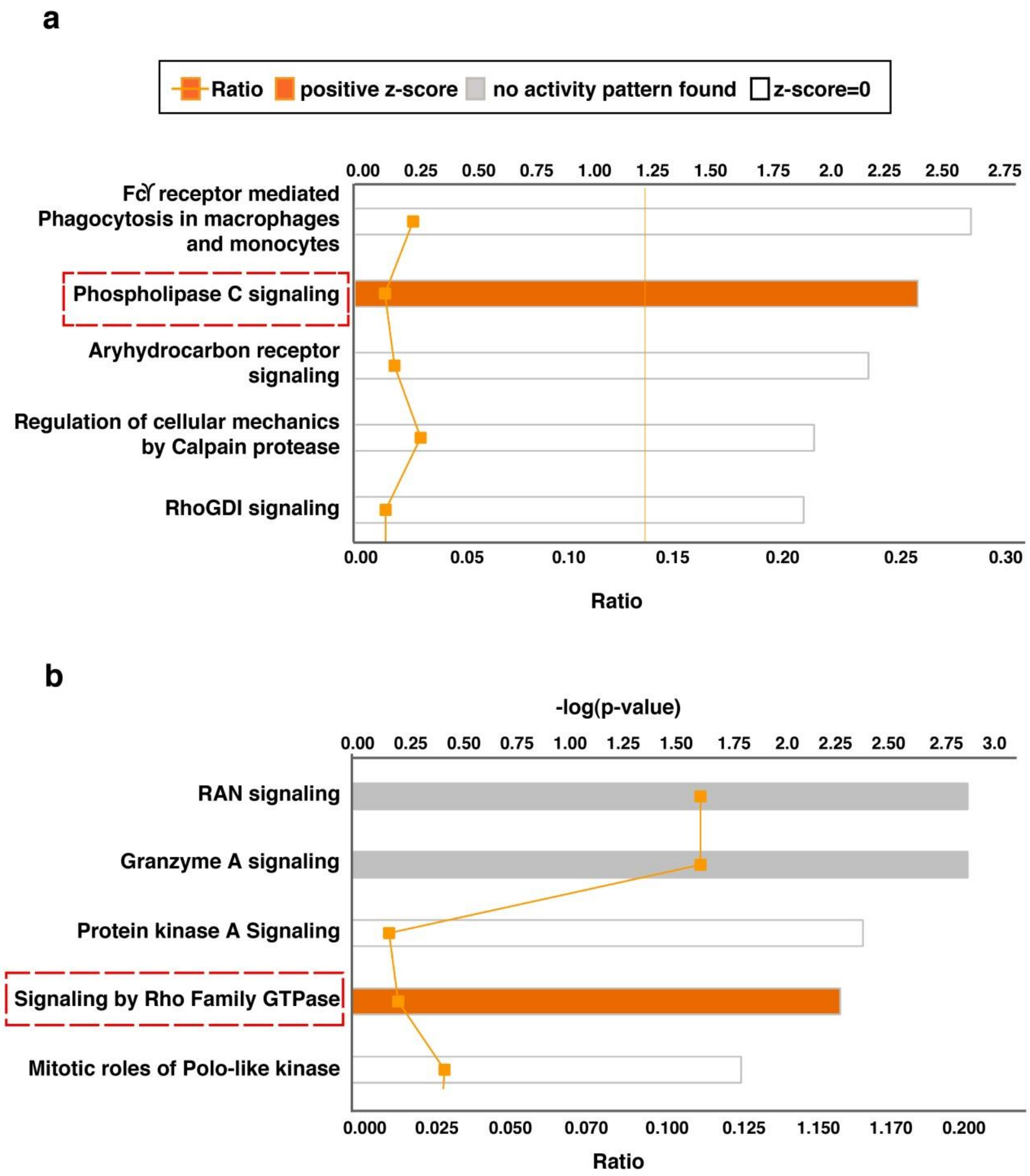

Supplementary Figure S3. Top five canonical pathways enriched by IPA in (a) luminal and (b) basal subtypes of bladder carcinoma.

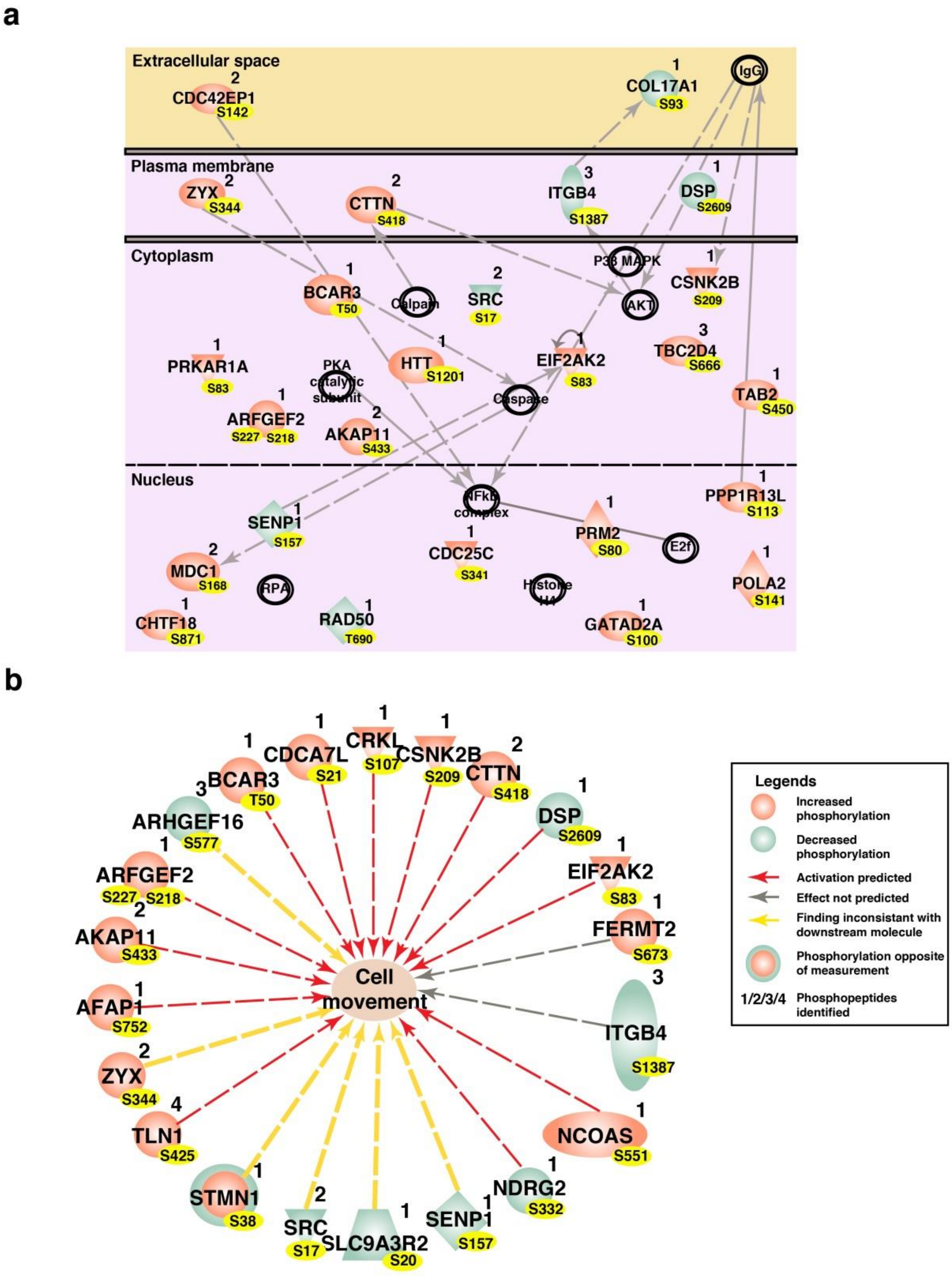

Supplementary Figure S4. (a) A global network interaction map of the differentially phosphorylated proteins in non-type subtype. Most enriched network in the aggressive subtype with an enrichment score of 46 (IPA). (b) Cell motility is an intrinsic characteristic of the non-type subtype of bladder carcinoma. Phosphoproteins involved in cell motility, as depicted from the IPA analysis.

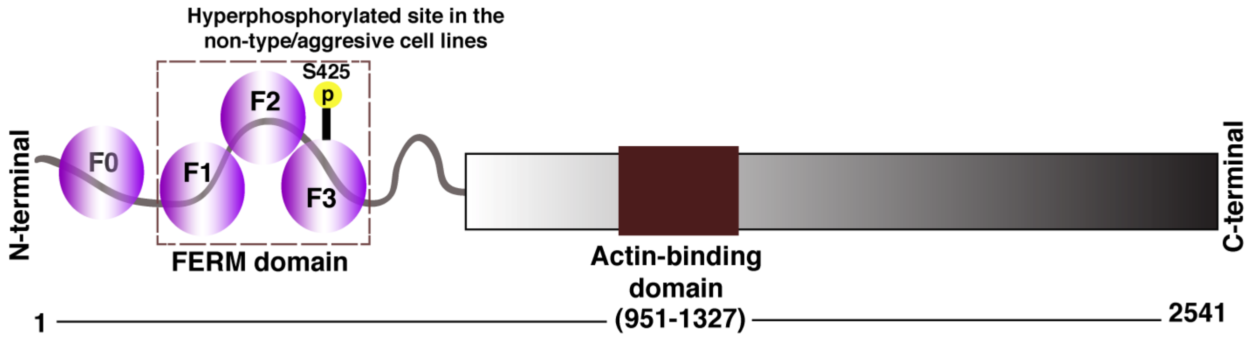

Supplementary Figure S5. Protein domain structure for TLN1. The domains of TLN1 and the significantly differentially phosphorylated sites (S425; head domain) in the non-type cell lines.

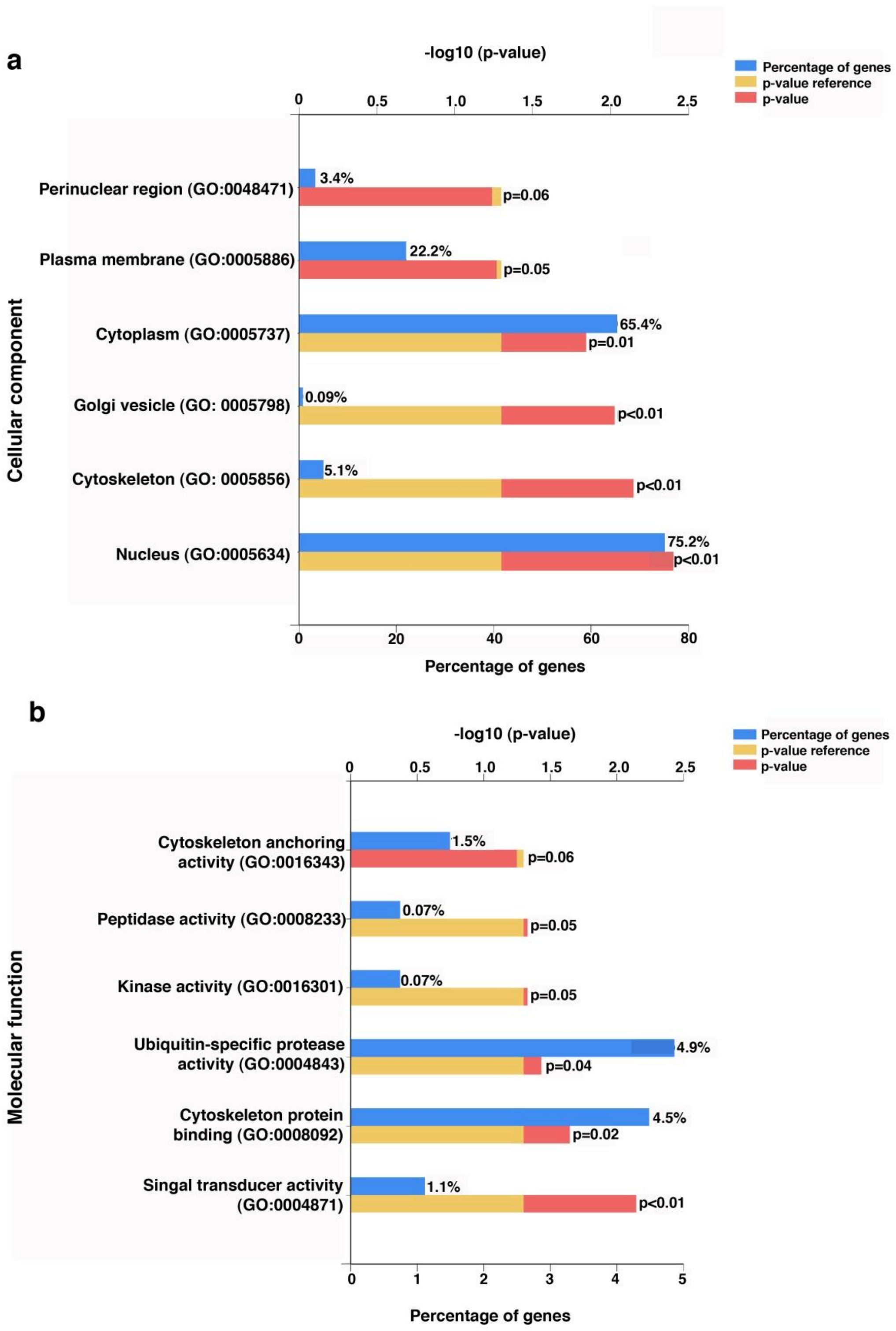

Supplementary Figure S6. Gene ontology enrichment of the differentially phosphorylated proteins in the non-type molecular subtype. Most of the proteins are nuclear proteins (75.2%). Proteins with kinase activity and signal transducer activity are also significantly enriched ($p \leq 0.05$). Data were analysed using FunRich.
